# Supplementary material for: Giving Families a Voice for Equitable Healthy Food Access in the Wake of Online Grocery Shopping
Source: Nutrients. 2022 Oct 19;14(20):4377. doi: 10.3390/nu14204377 (PMC9609455; doi:10.3390/nu14204377)
Supplement: Supplementary file 1 [file nutrients-14-04377-s001.zip › nutrients-1903734-supplementary.pdf]

SUPPLEMENTARY MATERIALS

Box S1. Interview Guide.

Guide for Focus Group Discussions (FGDs)

**PI:**	Angela Trude
**Study Title:**	Online Food Purchasing Patterns among Families Receiving SNAP
**IRB No.:**	HP-00090624
**PI Version Number/Date:**	Version 2, Jan 14th, 2021

A total of 5 focus group discussions (n = 5-10 in each) will be conducted with a community sample of SNAP-eligible families who have and have not purchased groceries online. The focus groups will gather participants' perspective and recommendations based on survey results. Focus group participants will complete surveys about food shopping habits, use of technology, familiarity with shopping online, and demographic characteristics before the focus groups. Focus groups will only occur if 3 or more participants are able to join the video-call; if only 1 or 2 participants attend, then an in-depth interview of the attending participants will be conducted by the facilitating researchers. Discussions will be one hour long, audio recorded, and each conducted virtually using Zoom. Discussion questions will further probe on attitudes, norms, and perceptions of online grocery shopping, perceptions of food access, impulse or unplanned buying, among other themes that may emerge in the discussions.

By checking this box, I understand that:

- This focus group discussion is part of a research study on grocery shopping
- The discussion will focus on grocery shopping habits, use of technology, and SNAP.
- If only two participants join the video-call, an in-depth interview with the facilitating researchers will be conducted instead
- It will last about 60 minutes and the discussion will be audio recorded to assist in transcription and support data analysis. Once discussion is transcribed, audio will be destroyed.
- All information obtained is confidential.
- My participation is voluntary and I can opt out any time

If you have any questions or concerns, please contact Angela Trude at atrude@som.umaryland.edu

Brief Qualtrics Survey

1. How many children are in the household? _________

2. Did you purchase groceries online in the past 6 months?
   - Yes
   - No

3. How many Internet-capable devices in your household?
   - None
   - 1-3
   - $\geq$ 4

4. How comfortable are you using technology devices, such as tablets, computers, and smart phones?
   - Very comfortable – I am fast and never need assistance
   - Somewhat comfortable – it takes me some time, but I can figure out
   - Not very comfortable – I usually request assistance
   - Not at all comfortable – I need others to help or do it for me

5. Does your internet support streaming services (e.g. Netflix) and video chat applications (e.g. Facetime or Zoom) even when multiple people in your house are online?
   - Yes, without any issues
   - Yes, but sometimes there are connectivity issues (e.g. slows down or videos freeze)
   - No, I don't have that capability
   - I'm not sure

6. How comfortable are you using Zoom?
   - Very comfortable – I use Zoom often and never need assistance
   - Somewhat comfortable – I have used Zoom multiple times and if I had issues I was able to resolve them on my own
   - Not very comfortable – I have only used Zoom a few times and I normally need assistance
   - Not at all comfortable – I am not familiar with Zoom

7. Monthly SNAP allotment
   - $1-250

$251-500
$501-750
$751-1,000

8. Food insecurity questions

In the last 12 months, were there times when the food for you and your family just did not last and there was no money to buy more?

Yes
Often
Sometimes
Almost every month

Please select whether the following statements were often, sometimes, or never true for you **over the 12 months.**

"That food that I bought just didn't last, and I didn't have money to get more."

Often
Sometimes
Never

"I couldn't afford to eat balanced meals."

Often
Sometimes
Never

In the last 12 months, did you or other adults in your household ever cut the size of your meals or skip meals because there wasn't enough money or food?

Yes
No

{If yes to above} How often did this happen?

Almost every month

Some months but not every month

Only 1 or 2 months

In the last 12 months, did you ever eat less than you felt you should because there wasn't enough money for food?

Yes

No

In the last 12 months, were you ever hungry but didn't eat because there wasn't enough money for food?

Yes

No

Focus Group Discussion Guide

Introduction:

Good (morning/afternoon) and thank you all for joining us today to talk about online grocery shopping. My name is [name] and I will be your moderator today. My colleague, [name], is also here and he will be taking notes for us.

Overview of the topic:

We are interested in learning more regarding how you feel about grocery shopping. The goal is for this to be interactive. We want you to have a dialogue with each other, so I will mostly just facilitate the conversation and ask questions.

Verbal Consent/Instructions:

- We will be recording the audio of our conversation today since we won't be able to capture everything in our notes.

- The recording will be kept safe and we won't be playing it for anyone outside of our research team.
- Also, your names will be excluded from the transcript of our conversation. Is everyone okay with our conversation being recorded? [*wait for response*]
- We want to remind you that your participation is voluntary. Although we want you to engage in the discussion as much as possible, if we ask any questions that make you uncomfortable, you do not have to answer it
- There are no right or wrong answers. There may be differing opinions or people who agree with one another. Both cases are fine. We are just interested in your perspective, whatever that may be. So, please feel free to share your point of view even if it is different from someone else's

Does everyone still want to participate?

Ground rules:

Before we start, I want to go over some ground rules just to make sure we are all on the same page and that we make the most out of our discussion today. How do you feel about:

- While someone else is speaking, we will have your mic muted. We want to minimize interruptions and make sure that everyone's points are heard.
- When you'd like to speak, please raise your hand (for us to see on the video) and we will invite you to unmute your mic.
- Although not required, we strongly encourage each participant to keep their video on to make this conversation more engaging.
- Lastly, we have a limited amount of time and we want to be respectful of everyone's schedule. I may need to interrupt you so I can make sure we cover all the topics today.

Are there any questions before we get started?

Okay, I'm going to start the recording now. (Record to the cloud)

Introduction: Purchasing Habits

I'd like to first begin by asking you all to:

1. Introduce yourselves by telling your name and something you like about grocery shopping and something you don't like about grocery shopping.

Attitudes and Perceptions towards Buying Grocery Online

Thank you all for sharing.

1. What are your thoughts about **online** grocery shopping?
   a. Probe: Why do you feel that way?

For mixed groups, with a show of hands, how many of you have shopped for groceries online before?

2. For those **who have**: Where have you bought groceries online?

3. What are the foods/beverages that you usually buy in-store but does not online?
   a. How about groceries that you get online but usually don't get in-store?
      1. Why do you think you buy more/less (of xx) online than in-store?
      2. Why do you think some people would do their groceries differently online compared to in-store?
   b. How would you compare the amount of money you usually spend when buying groceries online versus in-store?

4. For those **who haven't**: Do you know of any options for online grocery shopping where you live?
   a. Have you thought of using it?
   b. Why did you decide to not buy groceries online?
   c. *Note: Probe to try to get at how far in the process they went and what stopped them from buying groceries online*

5. **For SNAP FGD**: Some grocery stores allow you to use your EBT card as an online payment
   a. Do you know of a store that offer this service?
   b. Have you used your SNAP EBT card online? How was your experience
   c. How has the ability to use your EBT card online changed where you usually get your groceries?

6. Some online grocery services give you the option to picking up the groceries at the store, curbside pick-up, and/or home delivery.
   a. What do you think about these different options?
      i. What are your thoughts on the fees involved with each service?

ii. How about the timing of getting your groceries, from when you buy it to actually having your groceries with you?

7. Some online grocery services also provide a ready grocery list based on your previous buys. {*may share our screen to show an example*}?
    a. Probe: What are some examples of grocery items that come up on this list for you?
    b. Probe on benefits it may give: healthfulness? saving time? cost?
        i. What are the ways that this feature help you and your family?
        ii. When would this feature not be helpful to you and your family?

Barriers of Buying Online and Equity

1. What do you think are some of the drawbacks of buying groceries online? [Probe on the following]

a. How easy or difficult it is to pay for groceries online?
    i. How do you use coupons, find sales, or use different methods to pay for your groceries?
        a. Cost
            i. How would you compare the cost of groceries you buy online versus in-store?
            ii. What are your thoughts on the costs involved to get to the store (ie, your transportation to the store – money and travel time) and the costs of online grocery (delivery and service fees, cost of the internet/data)?

b. How would you compare the quality of meat and produce when buying online versus in-store?
    i. How do you feel about someone else selecting certain food items at the store for you?
    ii. Sometimes a grocery item is not available at the store and so it is replaced by another one. What do you think about the replacement?

2. Now, I want you take a few seconds to think about different groups of people in terms of race, education, socioeconomic background, and where they live (city or rural). Do you have some people in mind?
    a. How do you think grocery stores make it harder for them to get foods?
    b. Which groups of people may have a harder time getting to grocery stores and affording healthy foods?
        i. Why do you think they are excluded?
    c. How does where you live influence how and where you buy your groceries?
3. What are some ways that online grocery shopping makes it easier to access healthy foods?

a. Can someone think of ways that online grocery services may make it harder for some people to access healthy foods?vcxz

4. How does the SNAP EBT program help people get healthy foods?
    a. How does SNAP make it **difficult** for people to get healthy foods?

Additional services/features of online grocery shopping that might motivate you to use it.

1. We would like to hear your suggestions on how to make the online grocery shopping experience better. What are some online grocery shopping features that would motivate you to buy (more often) groceries online? [Probe on:]
    - Budgeting services
    - Convenience
    - Filters
    - Recipes
    - Shopping list builder

SNAP program

1. What, if anything, do you want to see added or changed in the SNAP program?

Other

2. Is there anything you would like to share with us today that we did not ask you?

Table S1. Emerging themes and selected quotes relating to the perceived causes of inequitable food access and most affected groups in the context of COVID-19 and online grocery shopping.

Causes of inequitable food access	Inequities intensified by COVID-19 pandemic
**Income distribution and high prices of healthy foods**<br>*"The pricing [of healthy food] is wrong. It's ridiculous that's causing a lot of obesity, because it's cheaper families that go out and get a bucket of chicken or a couple burgers at a fast food place where they are $1 or less than it is to get something fresh you know."* [Female, White/Caucasian, SNAP-participant]	**Perceived increase in prices of healthy foods**<br>*"Food prices have soared since the pandemic."* [Male, Black/African American, SNAP-participant]
**Low purchasing power and monetary resources**<br>*"And then you also have people that might not be as financially able to have a card that they can use to shop online. […] Like they don't even have a bank account […]"*[Female, White/Caucasian, non-SNAP participant]	**Pandemic exacerbated inequities in work and economic conditions**<br>*"So you have people who have lost their jobs, or because of COVID, and they're not receiving help from the government, from the state. Everything's at a standstill. What do they do? How do they feed*

	themselves? Where do they get the money for food? They can't work. They have small children who can't go to daycare because it's been closed. Can't go to school because they've either completely closed down schools or they're sending all the kids home to learn online." [Female, White/Caucasian, non-SNAP participant]
**Transportation limitations**<br>*"I think, like the transportation, if you live in an area where the bus doesn't move about there every half an hour, it is a problem. Or if there are places where the bus goes there once, twice during the day they don't go back, so that's tough if you live around that neighborhood and you don't drive."* [Female, Other race, non-SNAP participant]	**Increased risk of infection in public transportation**<br>*"[…] But also especially during COVID it's probably a lot harder to get to a store in the city because you have to worry about either walking or getting on a crowded bus or just being around more people in the city […]"*[Female, White/Caucasian, SNAP-participant]
**Internet and technology access**<br>*"Well, a lot of folks don't have computers or Internet service. […]They may be stuck on just everyday trying to survive to pay the bills. They don't have*	**Limitations with devices and social distancing**<br>*"And a lot of families in my area don't have the option to have technology, especially with COVID. A lot of the teens, they at least got electronic (.)*

money to shop online or that's not an issue with them. That's the least of their concerns, is doing stuff online." [Female, Black/African American, SNAP-participant]	*knowledge through school and our local library. And with schools closed and the library is closed, they're losing out on so much more in there. And they can't show their parents, because they don't have devices at home."* [Female, White/Caucasian, non-SNAP participant]
**Groups mainly affected by inequities in food access**	**Groups mainly affected during COVID-19 pandemic**
**Low-income/low-access areas and r ural communities**	
**Difficulty making healthy food decisions** *"Well, people in rural areas, but also people in urban areas too, like in bad, like impoverished neighborhoods, because it's harder for them to get the stuff that's healthy and the stuff that they need, […] But the stuff that's there for us is cheaper than anything, and when you got kids, you try to make the best decisions, but also an affordable decision so that it lasts."* [Female, Black/African American, SNAP-participant]	**Lack of availability of healthy foods** *"With COVID, my stores were out of so much that I didn't honestly know how we were going to make it, because I had to buy things that were triple the price, just so we would have something to eat versus being hungry. And it wound up being a lot of microwave stuff and a lot of not actual food that didn't last as long but was triple the price. And sometimes… every time somebody says "Oh, the COVID numbers are rising," we get the*

	same experience. So the stores are empty, the online stores are empty, and there's (..) there's not really any options, especially living in a small area." *[Female, White/Caucasian, non-SAP participant]*
**Older adults and people with disabilities**	
**Issues with accessibility** *"I think [online grocery] is really good. But I think they could do better with it to assist people, you know. Whether it's health conditions or just the elderly. The elderly do not get as much help, either order and pay the fees, or try to figure out how to come out and get their groceries. And sometimes their groceries are very heavy. So, then they're stuck with the walking up the stairs or having that option of just paying that small fee."* [Female, Black/African American, SNAP-participant]	**Problems with internet access, technology capacity (online grocery shopping) and increased risk of infection (in-store shopping)** *"I think, honestly, the elderly and disabled people would have probably the hardest time with online shopping and in store either way they go. Most older people aren't very tech savvy to be able to order online and they're vulnerable [to COVID-19 infection] if they go out into the public right now to go in store to grocery shop."* [Female, White/Caucasian, SNAP-participant]

Racial/ethnic diverse groups	
**Disparate food environments**	**Disparate impact of pandemic**
"My think is can you go into [affluent neighborhood] or can you go into [another affluent neighborhood] or something like that, and you see them people shopping at [dollar store chain]? No you don't. You don't see them people shopping at [dollar store chain]. They got their [full-size grocery chain], they got all that, but you come…and you come into an African American neighborhood or in a Latina neighborhood and where they're all flocking to family dollar with their food stamps to get food. Why? Why can't they have a store like that so they can get- they deserve fresh vegetables and organic things and they deserve it too!" [Female, Black/African American, SNAP-participant]	*"I know that COVID has hit the Black and Hispanic communities a lot worse than it has the white community. So, I feel like that it must be a lot scarier for someone of color or somebody living in a big city to know those facts, and still have to go out and do that, go out to the grocery store."* [Female, White/Caucasian, SNAP-participant]

Abbreviations: SNAP [Supplemental Nutrition Assistance Program]
Other races include Hispanic or Latino, Asian, Middle Eastern, American Indian, or Native Hawaiian.

Table S2. Emerging themes and selected quotes relating to the perceived role of Supplemental Nutrition Assistance Program (SNAP) and online grocery services in food inequities.

	**Help address inequities**	**Unintended consequences**
**SNAP**	**Improve food access**<br>*"It definitely helps you not have to stretch your dollar just to be able to afford some carrots or something. You know you're going to be able to specifically buy that healthy food with food stamps [SNAP] instead of deciding whether to buy diapers or food with money."* [Female, White/Caucasian, SNAP-participant]	**Limited amount of the SNAP benefit**<br>"[The negative part of SNAP is] *The limited amount that they give. Because again, before this pandemic hit, I was getting $132 month to feed three people. So, if you don't have enough to make it through that month, you gotta hit and miss and therefore that means you get food with preservatives in it. Because sometimes the healthier food tends to cost more."* [Male, Black/African American, SNAP-participant]
	**Expansion of the benefits in the pandemic**<br>*"One good thing now that I say about this pandemic and, as far as food goes, the state of Maryland has increased the amount of food*	**Purchase of prepared foods not permitted/ problems with storing and cooking foods**

stamps [SNAP] that they allot to people and families. Which means your families could actually buy better grades of food, more healthier foods." [Male, Black/African American, SNAP-participant]	*"Well me personally (...) they are going to give you food stamps [SNAP] but they don't ever think how I am going to get to the store, where am I putting this food at if I don't have a home, what am I doing with this food like I can't get hot food? (...)I gotta go to the market, every day, and just eat off the card every day okay,"* [Female, Black/African American, SNAP-participant]
**Expansion of the SNAP-OPP** *"It makes people be able to afford, afford not only…I mean afford healthy foods, I imagine that's true. And on top of it, this whole being able to buy things online has got to make things better for people in food deserts. If people know they can, I'm assuming most people know now that they can get their food online."* [Female, White/Caucasian, SNAP-participant]	**SNAP card use and stigma** *"Sometimes, it depends on the store, it depends on the area. Some corner stores in certain areas don't accept* [SNAP] *EBT card because they couldn't get approval, or someone scammed and caused a problem, complained, so they got shut down. So it is one less area for citizens of that community to access food. You need to come up with a way where you're not discriminated in any way shape or form, to where you can eat healthy foods for your family.*

		You know, no that is not asking for much. That is a basic need, food, you need food to survive." [Female, White/Caucasian, SNAP-participant]
**Online grocery**	**Food access and convenience**<br>*"You know if you're ordering them online and they're being delivered. Then you have no problem without having to worry. Like if you are in an area where you can't get to the grocery store, you can get the celery and the tomatoes and the lettuce and bananas delivered versus, if you had to take public transportation or something, then you gotta haul it home. If it's being delivered right to the house, it's a lot easier."* [Female, White/Caucasian, SNAP-participant]	**Lack of control over food selection**<br>*"I think a lot of people may have trouble with the fact that somebody else is hand selecting their fruit and vegetables. I don't think really there is anything else that online can do."* [Female, White/Caucasian, non-SNAP participant]
	**Specific groups**	**Fees and extra payments**

	"Online shopping is good for older people. Because my grandmother, I know, she orders from Safeway. She orders all her groceries online, so this is beneficial for people who can't get out and people who can't travel to get to the grocery store." [Female, Black/African American, SNAP-participant]	*"because if you're, even if you're on EBT or food stamps, do you have the extra money to tip these drivers when they come to you with the food? Probably not, you know."* [Male, Black/African American, SNAP-participant]

Abbreviations: SNAP [Supplemental Nutrition Assistance Program], EBT [Electronic Benefit Transfer]
